# Refractory lactotroph adenomas

**DOI:** 10.1007/s11102-023-01305-8

**Published:** 2023-03-16

**Authors:** Sandrine A. Urwyler, Niki Karavitaki

**Affiliations:** 1 Institute of Metabolism and Systems Research (ISMR), College of Medical and Dental Sciences, University of Birmingham, IBR Tower, Level 2, Birmingham, B15 2TT UK; 2 Centre for Endocrinology, Diabetes and Metabolism, Birmingham Health Partners, Birmingham, UK; 3 Department of Endocrinology, Queen Elizabeth Hospital, University Hospitals Birmingham NHS Foundation Trust, Birmingham, UK

**Keywords:** Prolactinoma, Aggressive prolactinoma, Malignant prolactinoma, Dopamine agonist resistance

## Abstract

A small subset of lactotroph adenomas is resistant to dopamine agonists (DA) and can also demonstrate aggressive or even malignant behavior. The implicated mechanisms are not clearly defined. Management can be challenging and requires a multidisciplinary approach. In DA resistant prolactinomas, switching to another DA could be the first option to consider. Further strategies include surgery and radiotherapy used alone or in combination. In cases of aggressive or malignant prolactinomas, temozolomide could be offered. Immune checkpoint inhibitors have been also recently proposed as an alternative approach. The place of other treatments (e.g., metformin, selective estrogen modulators, somatostatin analogues, tyrosine kinase inhibitors, inhibitors of mammalian target of rapamycin and peptide radio-receptor therapy) remains to be carefully assessed.

## Introduction

Dopamine agonists (DA) are recommended as first-line therapy in prolactin-secreting adenomas [1] leading to prolactin normalization and tumor shrinkage in the majority of the cases [2]. However, a small subset of tumors show resistance to these agents. The criteria defining resistance to DAs are variable in the literature making comparisons between different DAs and identification of predictors of response challenging [3]. Based on the Endocrine Society guidelines, DA resistance is defined as failure to restore normoprolactinemia on maximally tolerated doses of DA and failure to achieve 50% tumor shrinkage [1]. It should be argued as to whether both these criteria need to be fulfilled and if the presence of only one of them is indeed indicative of resistance to DA treatment. A further point deserving attention is whether the definition of resistance should include the use of the maximally tolerated dose of DA or a specific cut-off dose; the approach of clinicians on this varies and consistent criteria are lacking. Overall, resistance to bromocriptine has been reported in 20–30% of the cases and to cabergoline in 10% of them [1, 3].

## Mechanisms of resistance and aggressive behavior

The mechanisms responsible for DA resistance are not completely understood. Reduction of D2-receptors (D2R) has been postulated [1, 4], however, the binding affinity of the D2R has found to be unaltered. Additional proposed mechanisms include alterations downstream the D2R contributing to insensitivity to the inhibitory dopaminergic effect (e.g., decrease of the inhibitory G protein alpha subunit mRNA levels, changes in the cytoskeleton protein filamin A, disruption in the autocrine growth factor signaling mediated either by tyrosine kinase receptor ErbB3 or by nerve growth factor receptor, which modulates D2R expression) [4]. The transforming growth factor beta-1 (TGFβ1) has also been implicated with DA resistance offering a potential target for novel treatments [4]. Clinical factors associated with DA resistance include male gender, larger tumor size and invasiveness, and younger age at diagnosis [1, 3].

A smaller subgroup of prolactinomas can demonstrate truly aggressive behavior. The European Society of Endocrinology (ESE) guidelines define aggressive pituitary tumors as, radiologically invasive tumors which show an unusual rapid rate of growth or clinically relevant growth, despite optimal standard therapies (surgery, radiotherapy and conventional medical treatments) [5]. The prevalence of aggressive prolactinomas is unknown [5]. Pituitary carcinomas, defined by the presence of metastatic deposits, are rare (prevalence 0.2% of all pituitary tumors) and have poor prognosis [5]. Lactotroph carcinomas are the second most frequent pituitary carcinomas comprising 37.5% of pituitary carcinomas in an ESE survey [6]. Attention should be paid in patients with aggressive pituitary tumors and either site-specific symptoms (e.g., neck/back pain or neurological complaints) or discordant biochemical and radiological findings, or developing secondary DA resistance, as rarely malignant transformation can occur [2, 5, 7]. In these cases, screening for metastatic disease is recommended [5].

The combination of proliferation markers (Ki-67 and mitotic count) with tumor invasiveness have been suggested as prognostic markers of aggressive tumor behavior [8]. In addition, low expression of estrogen receptor α (ERα) and overexpression of vascular endothelial growth factor (VEGF) have been associated with lactotroph tumor aggressiveness. Further factors that have been proposed to be correlated with aggressiveness include expression of genes regulating invasion and proliferation [e.g., ADAM Metallopeptidase With Thrombospondin Type 1 Motif 6 (*ADAMTS6*), Collapsin Response Mediator Protein 1 (*CRMP1*), Pituitary Tumor Transforming Gene (*PTTG*)], expression of matrix metalloproteinase 9 and presence of chromosome abnormalities (e.g., loss of heterozygosity in chromosome 11) [8].

## **Management of resistant/aggressive prolactinomas**

### Changes in DAs

The first approach will be changing the DA brand if not taking cabergoline. In patients not responding to bromocriptine, change to cabergoline is advised [1] based on studies reporting normalization of prolactin in 100% of microadenomas and 79% of macroadenomas resistant to bromocriptine [9]. It has also been shown that cabergoline leads to notable reduction in tumor size even in patients with partial tumor shrinkage whilst on quinagolide [10], suggesting that switch from quinagolide to cabergoline may also be considered.

Second approach would be to maximize the dose of cabergoline to the maximal tolerated one for at least 3–6 months [5]. The maximum tolerated doses vary amongst patients and can be up to 12 mg/week [4, 9].

Notably, based on very limited available literature, switching to bromocriptine in cases of resistant to cabergoline prolactinomas may be an alternative choice leading to normoprolactinemia [10]. Nonetheless, this option would be anticipated to have very low chance of success.

### Pituitary surgery

Pituitary surgery is a further approach [11, 12]. The literature looking at the outcomes of surgical management in DA resistant prolactinomas is confounded by the inclusion of cases in which surgery was offered also due to DA intolerance or patient preference or presence of acute complications. Within these limitations, review of studies published between 2000 and 2015 has shown that transsphenoidal surgery leads to normoprolactinemia in 71–100% of microprolactinomas (with prolactin checked shortly after or within the first weeks following surgery) [11]. Nonetheless, recurrence of hyperprolactinemia has been reported between 0% and 50% of the cases, with this wide range attributed to the variable definitions of cure/recurrence, as well as to the different observation periods and dropout rates) [11]. Post-operative complications are low and new pituitary hormone deficits have been described between 0% and 6% of the patients [11]. Surgery in macroprolactinomas offers less optimal results with remission rates ranging between 32% and 60% in non-invasive tumors and reported 5 years relapse rates of 70% [13, 14]. In invasive macroprolactinomas, achievement of remission is not possible, whereas diagnostic prolactin values and presence of residuum post-operatively have been identified as factors associated with remission [13]. It has also been shown that partial resection of a DA resistant prolactinoma can allow prolactin normalization with a lower dose of DA offered post-operatively [13].

It should be also noted that surgical expertise is of major importance for the outcomes necessitating the performance of these operations by experienced pituitary surgeons [5, 15].

### Radiotherapy

Radiotherapy in DA resistant prolactinomas is mainly used as a part of a multimodal treatment approach, usually after surgery.

Review of data from 610 patients with prolactinoma offered stereotactic radiosurgery for various reasons including DA resistance, showed tumor control rates between 83% and 100% during monitoring periods 16–96 months; biochemical remission was achieved at a lower rate, reported between 16% and 83% in the different series (marginal dose 15–34 Gy) [16]. Based on published data of a very limited number of cases offered fractionated stereotactic radiotherapy, normal prolactin has been achieved between 0% and 40% of the cases during follow-up periods of 19 and 29 months, respectively [17, 18].

### Temozolomide and other management options

Temozolomide, an alkylating agent with lipophilic properties that crosses the blood-brain barrier, is considered as first-line chemotherapy for aggressive pituitary tumors and carcinomas [5]. In the ESE survey 2016, amongst 40 cases of lactotroph adenomas (25 aggressive adenomas and 15 carcinomas) were treated with this agent, 45% achieved partial response (tumor shrinkage > 30%), 5% had complete response with no tumor visible, 26% had stable disease (< 30% regression, but no more than 10% tumor size increase) and 24% showed progressive disease (> 10% tumor enlargement or new metastasis) [6]. In cases of temozolomide failure, evidence-based treatment is not available. A French cohort study on real-life efficacy of immune checkpoint inhibitor therapy on pituitary tumors showed some success for lactotroph carcinomas and to a lesser extent for aggressive lactotroph tumors [19]. Although the study relied on a very small number of cases, immune checkpoint inhibitors may be considered in patients failing to respond to temozolomide [19].

Other therapeutic options have been proposed for DA resistant prolactinomas, such as metformin, selective estrogen modulators, somatostatin analogues, tyrosine kinase inhibitors, inhibitors of mammalian target of rapamycin (mTOR), immune checkpoint inhibitors and peptide radio-receptor therapy [3, 19]. Data on their efficacy are very limited and a brief overview is provided in Table 1.

**Table 1:** Brief overview of alternative treatment options for refractory prolactinomas

Agent	Studies	Outcome
Metformin		
Metformin in combination to bromocriptine	Two case reports [20]	Reduction in prolactin levels in combination with tumor shrinkage in both cases. However, in the second case, tumor shrinkage was also associated with hemorrhage.
Selective estrogen receptor modulators (SERM)		
Tamoxifen/ Raloxifen	Four studies of 34 patients [21–24]	Tamoxifen: limited and inconclusive results. Raloxifen: limited data showing minimal decrease in prolactin levels. No data on the effect on tumor volume.
Somatostatin analogues (SSA)		
Octreotide LAR	Two case series with five and two patients, respectively [25, 26] Three case reports [27–29]	Conflicting results for treatment with octreotide LAR in addition to cabergoline.
Pasireotide	Two case reports [30, 31]	Conflicting results: biochemical control in two cases, tumor shrinkage in one and no effect on tumor size in the second case.
Tyrosine kinase inhibitors (TKIs)		
Lapatinib (EGFR/ErbB2 targeting TKI)	Prospective, phase 2a multicenter trial with four patients [32]	None of the patients achieved the primary endpoint (40% reduction in any tumor dimension). Three patients showed stable disease (two had 6% increase in tumor diameter and one had 16.8% decrease in tumor diameter; also, two of these patients had a 28% and 59% increase in prolactin levels). One patient withdrew after three months on Lapatinib due to progressive disease on imaging.
Inhibitors of mammalian target of rapamycin (mTOR)		
Everolimus	One case report [33]	44% reduction in prolactin levels and stable tumor size over one year.
Peptide radio-receptor therapy (PPRT)		
^177^Lu-DOTA-TATE, ^177^Lu-DOTA-TOC, ^90^Yttrium-DOTA-TOC, ^111^In-DTPA-octreotide	ESE survey including five patients with aggressive lactotroph tumor [34]	Four patients had progressive disease with tumor growth, one patient had partial reduction of tumor size 84 months after treatment with ^111^In-DTPA-octreotide.
Immune Checkpoint Inhibitors (ICI)		
Ipilimumab/Nivolumab	Cohort study with two lactotroph carcinomas and four aggressive lactotroph tumors [19]	Out of two lactotroph carcinomas, one showed partial response with normalization of prolactin and one progressed. Out of four aggressive lactotroph tumors, one had stable disease with partial biochemical response and three showed progressive disease.

## Conclusions and future perspectives

DA resistance can be managed with various options used alone or in combination. In depth understanding of the pathogenetic mechanisms implicated in this phenomenon could open new therapeutic avenues. Aggressive or malignant prolactinomas remain challenging scenarios requiring multidisciplinary approach; in these cases, temozolomide is the first chemotherapeutic agent of choice, whereas immune checkpoint inhibitors may be a further potential option. The place of other medical treatments remains to be carefully assessed.
